# Thermal Sources of Errors in Surface Texture Imaging

**DOI:** 10.3390/ma13102337

**Published:** 2020-05-19

**Authors:** Karol Grochalski, Michał Wieczorowski, Paweł Pawlus, Jihad H’Roura

**Affiliations:** 1 Division of Metrology and Measurement Systems, Institute of Mechanical Technology, Poznan University of Technology, Piotrowo 3, PL-60965 Poznan, Poland; michal.wieczorowski@put.poznan.pl; 2 Faculty of Mechanical Engineering and Aeronautics, Rzeszow University of Technology, al. Powstańców Warszawy 12, PL-35959 Rzeszow, Poland; ppawlus@prz.edu.pl; 3 IRF-SIC Laboratory, Department of Mathematics, Ibn Zohr University, Nouveau Complexe Universitaire, 80000 Agadir, Morocco; jihad.hroura@gmail.com

**Keywords:** contact profilometry, surface topography, thermal disturbance, thermal expansion, thermal chamber

## Abstract

This paper presents the influence of thermal phenomena on areal measurements of surface topography using contact profilometers. The research concerned measurements under controlled and variable environmental conditions. The influence of internal heat sources from profilometer drives and their electronic components was analyzed. For this purpose, a thermal chamber was designed and built. Its task was to maintain and control environmental conditions and, at the same time, separate the profilometer from external disturbances. Heat sources and temperature values for elements and systems were determined. It further enabled for the calculation of the displacements in axes as a function of temperature. The largest displacement in the probe due to internal heat sources for the considered cases occurred in the *X*-axis direction. Its value reached 16.2 μm. However, the displacement in the probe in the *Z*-axis direction had the greatest impact on the measured surface topography. These displacements for a thermally unstable profilometer reached 7.9 μm in Z, causing results even 90% greater than in the case of a device without such problems. The time after which a proper topography measurement can be started was also determined basing on obtained data. This time for tested profilometers was between 6 and 12 h. It was found that performing thermal stabilization of the profilometer significantly reduced surface irregularity errors. The stabilization time should be determined individually for a specific type of device.

## 1. Introduction

The improvement in the quality of manufacturing processes of machinery and equipment parts is directly related to the development (advancement) of methods to control manufactured products. This is associated with the simultaneous development of quality control departments and the use of more and more advanced measuring devices. One of the most important factors determining the quality of a product is the surface structure of the produced element. It is a consequence of production methods and machining [1].

An important issue in the assessment of the structure condition is broadly understood surface irregularities. They are one of the basic features determining the quality of manufactured parts that also affect their performance, durability, and exchangeability in mechanical engineering [2]. The geometric structure of the surface (GSS), known also as surface texture, is the set of all hills and dales on the surface.

A surface irregularity consists of waviness, roughness, and shape deviation. These components can be assigned to three main groups of deviation scale:

- large scale—shape deviation,
- average scale—waviness,
- small scale—roughness.

There are many methods and devices for surface topography analysis [3], including tactile and optical ones [4]. The stylus method is the oldest yet still the most often used in industry. In this method, the surface texture is often analyzed on the basis of cross-sections—surface asperity profiles. However, three-dimensional (3D) surface analysis provides more important information. There are also sophisticated methods of filtration, enabling the separation of particular components, e.g., the component of a shape deviation or waviness, which, as a result, enables the roughness analysis. They often reach even more and more multiscale analysis for different purposes [5,6].

In contact methods, a measurement is carried out using a diamond measuring tip that moves over the measured surface. These methods are well known, but they are also sensitive to some interfering factors, such as vibrations, thermal phenomena, and geometrical errors of the measuring tip, including the rounding radius and local changes in the tip. Errors in roughness measurements resulting from the tip geometry have been the subject of a number of studies, e.g., performed by Elewa and Koura [7], Bodschwinna [8], Trumpold and Heldt [9], Smith and Chetwynd [10], and Anbari et al. [11]. The shape of the measuring tip changes the actual geometry of asperity. An additional source of error in spatial measurements is the linear displacement of the measuring tip in one axis of the profilometer, causing path synchronization error [12,13], as well as too high a velocity of the tip, causing the detachment of the tip from the measured element [14], for which various options were investigated [15].

Modern-quality control requires the use of devices that correctly return information about the measurement of the selected quantity while maintaining the required accuracy parameters. When the measurement is performed using typical measuring devices, solutions are sought to eliminate the influence of disturbing factors after the test. This is one of the issues related to information-rich metrology [16]. The data set covering many aspects of the measurement, e.g., environmental factors, device status, and signal processing algorithms, allows the most advantageous analysis of the obtained values and their possible correct interpretation.

## 2. Problem Statement

There are generally a lot of sources of errors in surface topography measurements using optical and tactile techniques [17,18]. In optical techniques, they are mainly connected with non-measured points [19] or sampling intervals [20], in stylus—with the geometry of a probe tip or vibrations and noise. Their impact is described in the references, e.g., work by Haitjema and Morel [21]. In the case of profilometric measurements, this is often associated with the way the environment affects the measuring device. Thermal disturbances are often not considered as an important source of error, which, particularly in the case of 3D measurements, is not true. They may have a form of changes in amplitude and/or frequency. Research on this type of disturbance was carried out, among others, by Miller et al. [22] and Krawiec et al. [23].

When measuring a single profile, provided the time of temperature change is long compared to the measurement time, low-frequency temperature changes do not influence the measurement results. In the case of long-term spatial measurements with a large number of measuring probe traverses, the influence of temperature changes on the measurement system can be significant, particularly when the temperature change occurs directly during the measurement procedure. According to the thermal expansion law, both the specimen and the measuring device undergo geometrical deformations in three dimensions.

The most frequent source of thermal errors is a commonly used, two-step air conditioning (cooling) system. Such a system turns on when the upper temperature limit is reached and turns off when the lower threshold is achieved. Such an operation of the air conditioning system is usually periodic. It also causes the direct influence of cold air (gas) on a device, which may, in turn, result in different temperature gradients. Zhou et al. investigated these phenomena in their work [24]. 

Furthermore, there are also internal thermal disturbances coming from electronic components, drives, and friction of the moving elements. Disturbances of these types are asymptotic and decrease with time during stabilization of the profilometer. The temperature stabilization process, which may take several hours, should be performed before the beginning of the measurement. Regardless of the nature of the disturbance, thermal phenomena cause errors in the image of the surface and values of parameters, calculated from the measured surface.

Currently, no thermal diagnostics and disturbance compensation are used in practical solutions for contact profilometers; they are carried out only for coordinate measuring machines (CMMs).

A way of compensation of certain errors related to the device geometry when a coordinate measuring technique is involved was proposed by Zha et al. [25]. Errors related to both displacement and deformation of the device components are most often associated with thermal expansion of the device. Thermal errors may also cause residual stress, particularly when construction elements are designed as multilayer ones [26]. Usually, measurements of deformation of this type are performed simultaneously with infrared thermal diagnostics. The methodology of thermal diagnostics of CMMs is presented in the works of Abdulshahed et al. [27], as well as Chenyang et al. [28]. Hao et al. [29] and Muniz et al. [30] also worked on similar issues related to thermal errors. On the contrary, Schwenke et al. tried to compensate these errors [31]. A similar attitude can be found in a publication by Ma et al. [32]. Sladek et al., on the other hand, were modeling the uncertainty changes caused by temperature for CMMs [33]. There are different additional devices used for calculating errors. The use of laser interferometry in deformation measurements of the CMM structure has been discussed by groups directed by Balsamo [34] and Stejskal [35]. Furthermore, the use of thermometry and laser interferometry is also applicable to other types of numerically controlled machines and devices, which makes their application much more versatile. The research concerning this topic has been described by Enming et al. [36], Lo et al. [37], and Miao et al. [38]. 

The recognition of issues related to temperature changes during measurements causes the need for compensation and thermal conditions control. The first attempts were made by Baird quite a long time ago [39]. Kruth et al. [40] presented the compensation of Thermal Errors on CMMs, dividing them into static and transient ones. Similar works for more generally considered devices were made by Sartori and Zhang [41]. Ge and Ding elaborated a method for thermal error control for precision parts of machine tools [42]. This method was based on the principle of thermal deformation balance. Tang, Xu, and Wang also conducted analyses in order to predict thermal deformations and the impact of environmental conditions on the measuring device using a neural network [43], while Milov et al. created an algorithm and software to identify errors in measuring equipment during the formation of permanent joints [44].

After analyzing the literature, it was decided to carry out the research on the influence of thermal disturbances on the surface asperities using contact profilometers, which will eventually enable compensation of this effect. Additionally, the authors intended to determine the time, after which thermal and geometrical stabilization will take place. The topic is an important issue as it is generally believed that there is no thermal influence on performance of a profilometer, which is commonly used for tactile (and not only) surface topography measurements.

## 3. Materials, Methods, and Results

The research on the influence of the internal heat sources of a profilometer on the expansion of its structure and elements was based on two types of measurements. First, a static measurement was made in which the measuring head of the profilometer remained stationary. Second, a dynamic measurement was performed, in which surface topography points were collected (measuring head was moving).

### 3.1. Static Measurement

Test methodology of the static measurement procedure (i.e., measurement without movement of a measuring head) was based on temperature verification of the profilometer components using two independent methods. The temperature measurement started with the moment the device was turned on (with no movement of the measuring probe in direction of *X*-axis). The power supply influenced the heating of electronic components, drives, and other heat-generating parts placed inside the profilometer structure. Simultaneously to the temperature measurement, the displacement of the measuring head was investigated using a laser interferometer. Temperature values were correlated with displacements, which allowed for the determination of the resultant thermal expansion coefficient of a particular element.

The first method of measuring temperature involved the use of semiconductor temperature sensors DS18B20+ (Maxim Integrated Products, Sunnyvale, CA, USA) localized as close as possible to the drives of individual axes of the profilometer. Sensors allowed to monitor changes in temperature as a function of time during the heating of the profilometer from the moment the device was turned on. A sensor of this type has a measuring range from −55 °C to +125 °C and a maximum permissible measurement error of ±0.5 °C in the measuring range from −10 °C to +85 °C.

The second method of temperature measurement involved the use of a diagnostic thermal imaging camera FLIR T620 (FLIR Systems, Inc., Wilsonville, Oregon, USA) which enabled detection of heat sources (profilometer components) in the entire system, presented in the form of a colored thermal map. The thermal imaging camera provided possibilities of monitoring the temperature distribution over time on all the profilometer surfaces and measuring the temperature in a certain point on a surface.

The use of the thermal imaging camera enabled the collection of more information about the thermal condition of the profilometer and very precise determination of heat sources.

The spatial (geometric) resolution of the T620 camera in the sense of the instantaneous field of view (IFOV) was 0.62 mrad and the thermal resolution in the sense of noise-equivalent temperature difference (NETD) was less than 0.05 °C. The maximum permissible measurement error of the camera was equal to ±2 °C or ±2% of the temperature readings (the greater of these two values is taken as the respective value).

**Figure 1:**
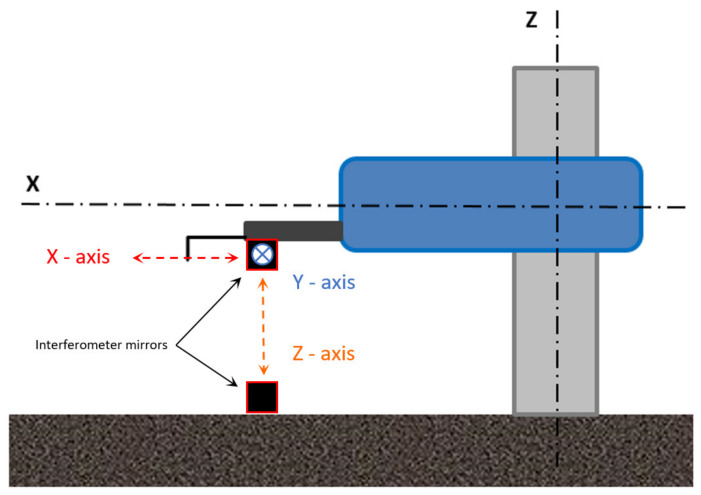
Scheme of the positions of interferometer mirrors for measuring displacements of *X*-, *Y*-, and *Z*-axes of profilometer, being under the influence of internal heat sources.

During testing of the profilometer, a laser interferometer LASERTEX LSP30-3D (Lasertex Sp. z o.o., Wrocław, Poland) with a measurement resolution of 0.1 nm was used. Its task was to measure the displacement of the measuring head, caused by changes in the geometrical dimensions of the device structure due to temperature variations from internal heat sources. The reference mirror (stationary) of the interferometer was located on a granite slab. The mobile mirror was attached to the fixing holder of the measuring head of the profilometer. The measurement head displacement was performed for each axis of the profilometer while maintaining the same experimental conditions. A similar method of determining geometrical deviations for coordinate measuring was reported by Hemming et al. [45], Echerfaoui et al. [46], and Schwenke et al. [47]. A scheme of the measuring setup is shown in Figure 1.

Research on the influence of internal heat sources was carried out on three contact profilometers, differing from each other in design and type of drives used for particular axes: T8000 (A), TOPO L50 (B), and S8P/PRK (C). The same measuring equipment settings and the same measurement methodology were used for each of these devices. During testing of the profilometers, the greatest attention was paid to the measurement of the elongation of the Z column. This was due to the direct possibility of a thermal deformation of the column affecting the correctness of the spatial imaging of surface topography.

**Figure 2:**
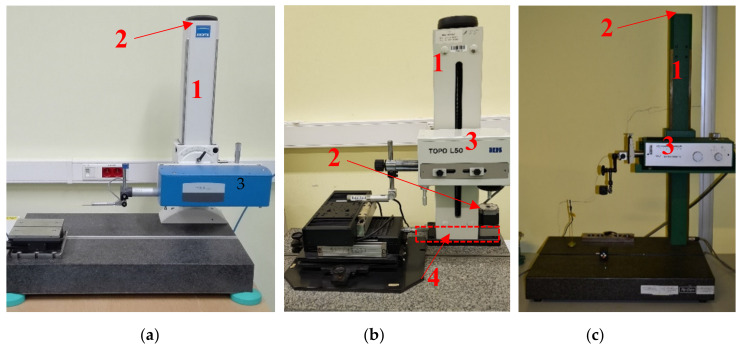
View of tested profilometers: 1—column, 2—drive Z, 3—traverse unit, 4—base. (**a**) Profilometer Hommel T8000; (**b**) Profilometer TOPO L50; (**c**) Profilometer Perthen S8P/PRK.

**Figure 3:**
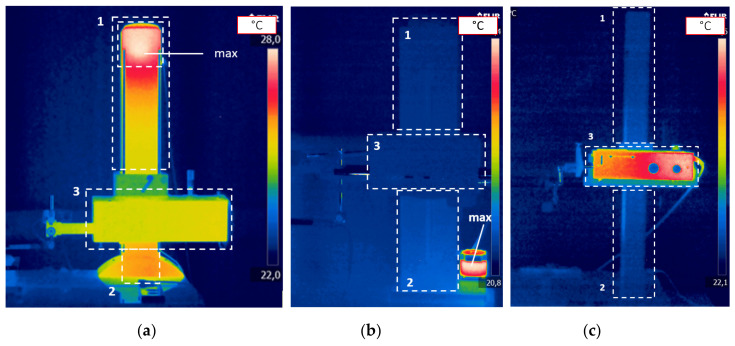
Distribution of thermal fields on the surface of profilometers: 1—part of the column above the traverse unit X, 2—part of the column below the traverse unit X, 3— traverse unit X. (**a**) Profilometer Hommel T8000; (**b**) Profilometer TOPO L50; (**c**) Profilometer Perthen S8P/PRK.

The distribution of thermal fields on the surface of each device after thermal stabilization, as well as the location and type of internal heat sources, is presented in Figure 2 and Figure 3, respectively. The thermal stabilization state of the device is based on the criterion for which the change in temperature values in the measuring points (column of the profilometer, fixtures of drives, and electronic components) during 3 h does not change more than 0.5 °C. This criterion was adapted from other research conducted for thermal stability for machine elements performed using the same temperature measuring devices [48].

**Figure 4:**
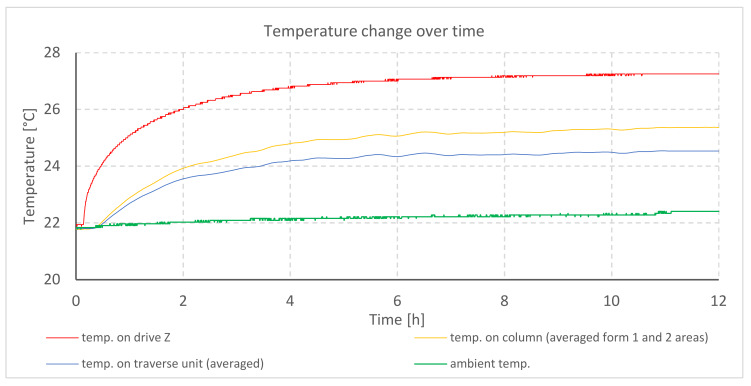
Temperature changes during heating of profilometer A drives.

**Figure 5:**
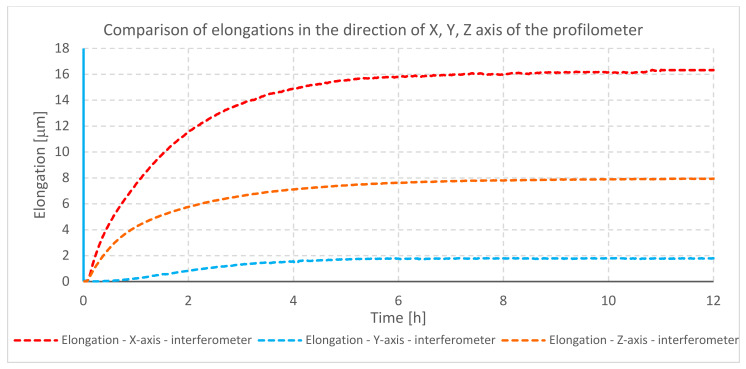
Comparison of elongation of individual axes of profilometer A during heating of its drives.

The temperature changes during testing of profilometer A are shown in the graph (Figure 4). The elongation of mechanical components for individual axes is illustrated in the next graph (Figure 5).

The graph (Figure 4) shows a rapid increase in the temperature of the stepper motor located in the Z column. Thermal stabilization of the drive and of the rest of the column can be observed after 6 h, counting from the moment the profilometer was turned on. The Z column increased its temperature by 4.3 °C and the traverse unit of the *X*-axis by 2.4 °C. At the same time, the growth of ambient temperature reached 0.4 °C.

The graph (Figure 5) shows displacements in the direction of individual axes due to elongation. The biggest change in the position during heating of the profilometer was observed in the direction of the *X*-axis. This is caused by a closed design of the traverse unit (*X*-axis), location of electronic modules inside it, and, due to this, practically no heat dissipation. The temperature inside the traverse unit directly affects the moving element of the measuring probe (pick-up), as well as the intermediary elements causing thermal expansion of all mentioned components. The smallest displacement was observed in the direction of the *Y*-axis of the profilometer. This is due to the design of the device. The traverse unit and its thermal deformability mostly affect moving parts and long elements (in accordance with the law of thermal expansion). There are no components in the *Y*-axis that can change their position, and the cross-section of the *Y*-axis has the smallest dimensions among all considered cross-sections (cross-section of the Z-column and traverse unit X).

**Figure 6:**
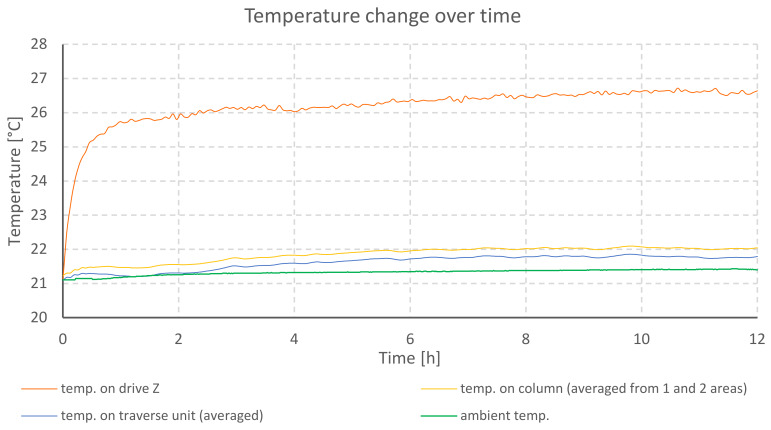
Temperature changes during heating of profilometer B drives.

**Figure 7:**
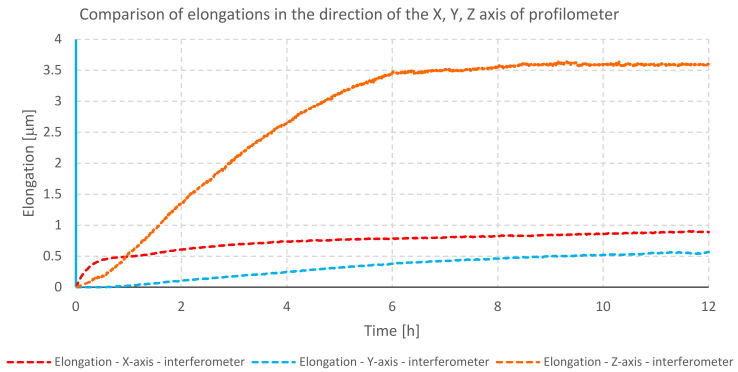
Comparison of elongation of individual axes of profilometer B during heating of its drives.

The temperature changes during testing of the profilometer B are shown in the graph (Figure 6). The elongation of mechanical components along individual axes is illustrated in the next graph (Figure 7).

The graph (Figure 6) also shows a rapid increase in the temperature of the stepper motor located in the Z column. The temperature becomes stable after 2 h and increases 4.87 °C. However, the real thermal stability can be observed after 6 h from the moment the profilometer was turned on. A difference of 5.34 °C from the initial temperature was achieved. The Z column increased its temperature by 0.7 °C and the traverse unit of the *X*-axis by 0.4 °C. At the same time, the growth of ambient temperature reached 0.2 °C. The location of the drive outside the body of the profilometer limits the thermal influence on other components. Still, some heat is distributed through the metal base connecting the column with the drive.

The graph (Figure 7) illustrating the displacement of the measuring probe presents changes in position due to elongation, during the heating of the profilometer. The largest displacement reaching 3.6 μm was observed in the direction of the *Z*-axis. It is caused by heat transfer from the stepper motor located in the Z column, which is mounted on a steel connecting plate, which is attached to a granite plate and to the base of the column. The displacement of the measuring probe in the *X*- and *Y*-axes shows a much smaller value that does not exceed 1 μm. These displacements can be a consequence of thermal interaction of individual components occurring in particular axes, as well as an effect of Z column deformation.

**Figure 8:**
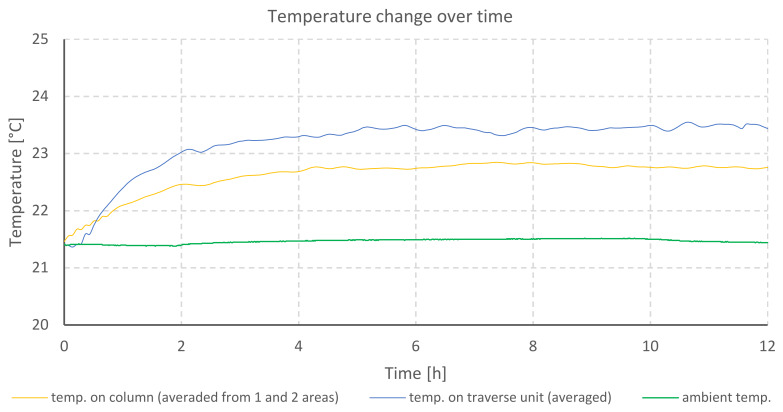
Temperature changes during heating of profilometer C drives.

**Figure 9:**
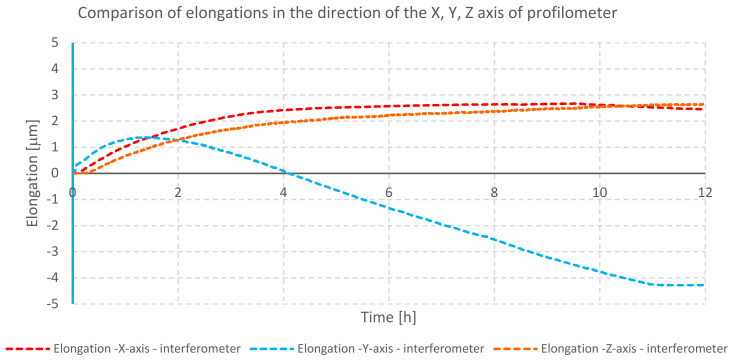
Comparison of elongation of individual axes of profilometer C during heating of its drives.

The temperature changes during testing of the profilometer C are shown in the graph (Figure 8). The elongation of mechanical components toward individual axes is illustrated in the next graph (Figure 9).

The temperature changes presented in the figure (Figure 8) show that the thermal stabilization of the traverse unit is reached about 4 h after switching the device on. The maximum temperature was recorded on the traverse unit X and was equal to 23.5 °C. The temperature increase of the Z column did not exceed 1 °C. The measurement was made in stable thermal environment conditions.

In the case of this device (profilometer C), column Z was not heated, which can be observed in the thermal image (Figure 3). For this reason, the temperature (drive Z) was not represented in the graph (Figure 8). It was measured by means of the semiconductor sensor located inside the housing, yet the readout was the same as ambient temperature. No heating effect is caused by a DC motor used to move the whole traverse unit in the Z direction. This kind of motor does not generate heat when the traverse unit is not moving vertically in contrast to stepper motors, where switching on the power supply already generates heat (as it was in profilometers A and B). 

**Figure 10:**
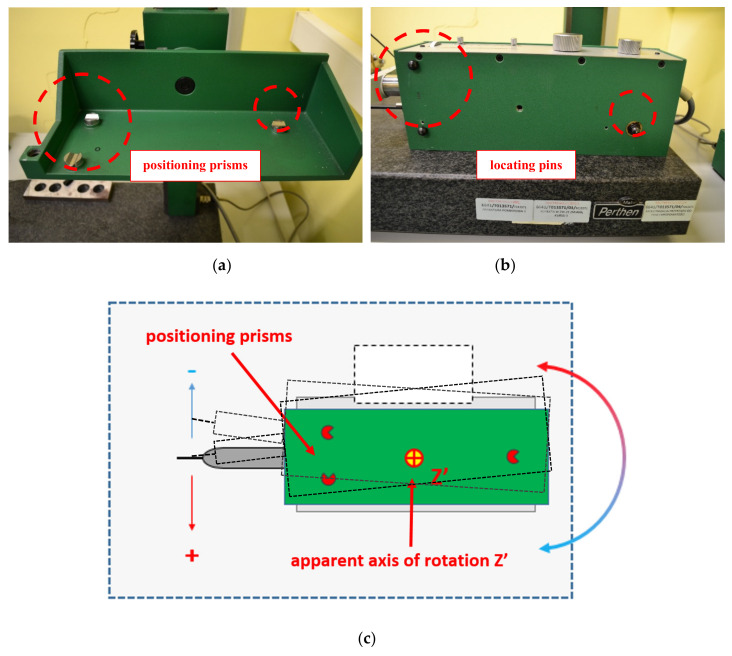
View of the supporting structure of X traverse unit of profilometer C: (**a**) Traverse unit cradle with positioning prisms marked; (**b**) traverse unit of the profilometer with locating pins marked; (**c**) deflection of the measuring probe of profilometer C relative to the apparent axis of rotation Z’.

The results presented in the graph (Figure 9) show the change in the position of the measuring probe during the heating of the profilometer caused by its internal heat sources in relation to the starting point. The values of displacements in the X and Z are smaller than 3 μm and monotonic. The measuring probe shows the largest changes in position during the heating of the device in the direction of the *Y*-axis in a characteristic non-monotonic way. This situation is not typical and is caused by uneven heating of the component and its location on the positioning prisms (Figure 10a,b), which enabled displacement associated with the rotation around the apparent axis Z (Figure 10c).

Expanding elements—beginning from the location of the *X*-axis drive—cause this part of the traverse unit to expand and move on the positioning prism. The heat is distributed to the remaining part of the unit. This causes elongation due to thermal expansion and, consequently, displacement on the next prisms and rotation around the apparent *Z*-axis.

**Figure 11:**
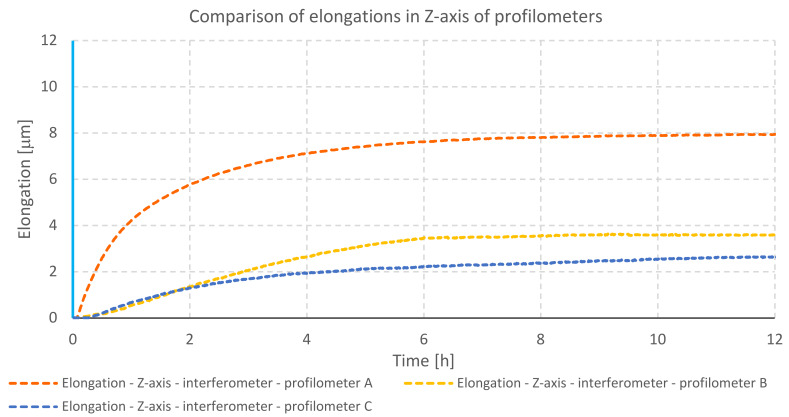
Comparison of elongation in *Z*-axes of profilometers during the heating of drives—internal heat sources.

Data collected from each test were analyzed and presented in the form of a graph (Figure 11). The displacement in the measuring head in the *Z*-axis of the tested profilometers can have a direct impact on the correctness of spatial imaging of surface topography. Further analysis contained deformation of a column.

Profilometer A has the highest *Z*-axis elongation value equal to 7.9 μm. Profilometer B has elongated by 3.58 μm, whereas profilometer C has elongated by 2.61 μm.

In general recommendations given by many manufacturers, it is suggested to turn the device on about 15 min before a measurement can be taken. Time determined experimentally, after which the 3D measurement of surface topography could be initialized, significantly varies from that information. It depends on the particular device and is about 6 h from the moment of turning the profilometer on (according to previously adopted criteria). After this time (in the case of tested devices), the temperature of the whole systems becomes stabilized, and no significant changes in geometric dimensions are expected.

In order to eliminate additional disturbances related to thermal aspects caused by, e.g., friction or traverse unit operation, it is recommended to perform a preliminary measurement, before starting a topography measurement. During this preliminary measurement, its time and conditions should be the same as during the real measurement. The only difference is a measuring tip that does not need to be in contact with the tested surface.

Based on the above presented results, it was found that profilometer A is characterized by the greatest susceptibility to the influence of internal heat sources. Therefore, further research should be concentrated on that device.

### 3.2. Dynamic Measurement

**Figure 12:**
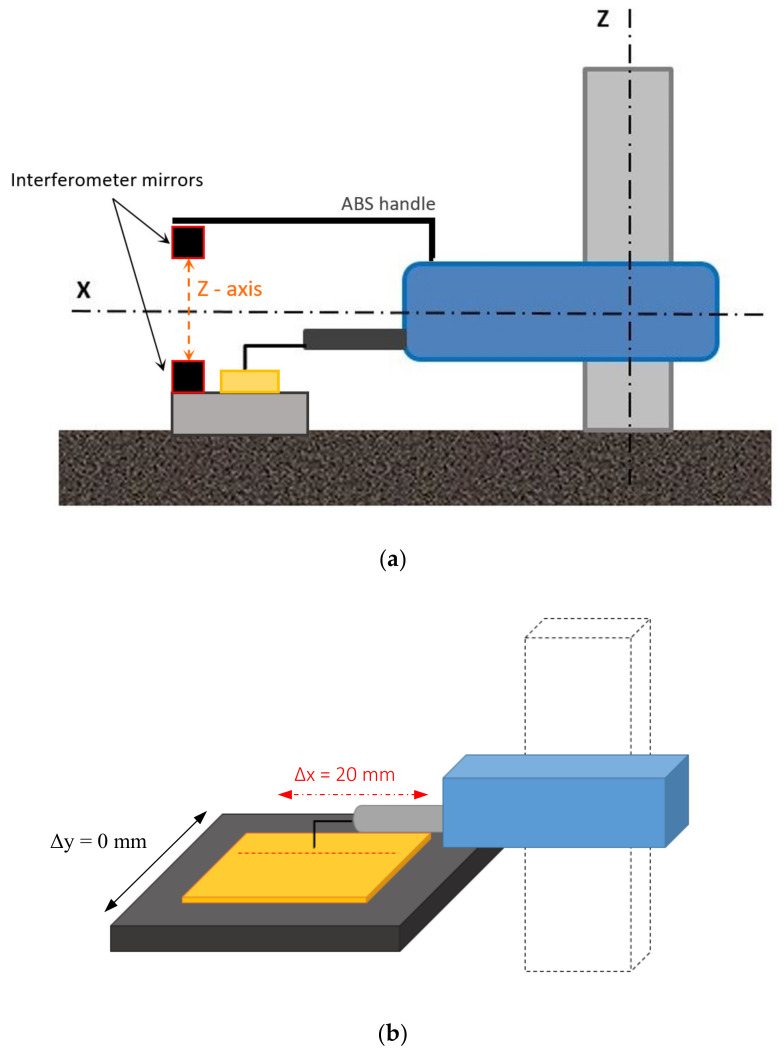
(**a**) Scheme of the interferometer mirrors during measurement of displacements in *Z*-axis; (**b**) graphic representation of measurement methodology.

The research methodology of the dynamic measurement (measurement with movement of a measuring probe), similar to the static measurement, was based on monitoring the temperature of the profilometer components using two independent methods. These methods involved using temperature sensors located in places of heat sources occurrence and using a thermal imaging camera. The temperature measurement began from the moment the device was turned on and surface topography measurement (scanning the measured surface with the measuring tip) started. Turning on the power supply of the device caused passive power consumption, while movement of mechanical elements resulted in the heating of profilometer components located inside its structure, causing an expansion of the whole device structure. The increase in temperature was correlated with results of the simultaneous measurement of the distance between the measuring probe and the base on which the tested specimen was placed. The distance measurement was performed using a laser interferometer. One of the mirrors (reference one) was placed on a sliding table. The second mirror was placed on a rigid ABS (acrylonitrile butadiene styrene terpolymer) handle, attached to the housing of the device (Figure 12a). The distance between the measuring head and the measured specimen was additionally verified during the measurement by reading the position of the measuring probe of the profilometer in relation to the sample. Information about the current position of the probe is registered and can be displayed in dedicated software.

The measured element was a standard optical flat—a parallel glass plate with known surface parameters and a flatness defined by the λ/20 parameter. In order to determine the influence of internal heat sources on the fidelity of surface topography imaging, the same profile was measured 600 times (to avoid influence of geometrical errors, the y table movement was switched off, Δy = 0). Then, the image of a surface was created from these profiles. This test, in the case of nearly ideal measuring conditions, was intended to provide an undisturbed, straight transverse profile from the generated surface. The measurement principle is presented in the figure (Figure 12b). 

Before the beginning of the measurement, the measuring probe was levelled in relation to the plate being measured. The height deviation of a 20 mm-long distance from the starting point to the end point did not exceed 0.1 μm.

Two types of tests of the dynamic measurement were performed. The first involved measurement of the surface topography from the moment the device was switched on. During that measurement procedure, the profilometer and its components were heating up.

The second test of the dynamic measurement involved measurements after thermal stabilization of the profilometer. The measurement was made on the same surface (the measured specimen was fixed and the table motion was turned off).

**Figure 13:**
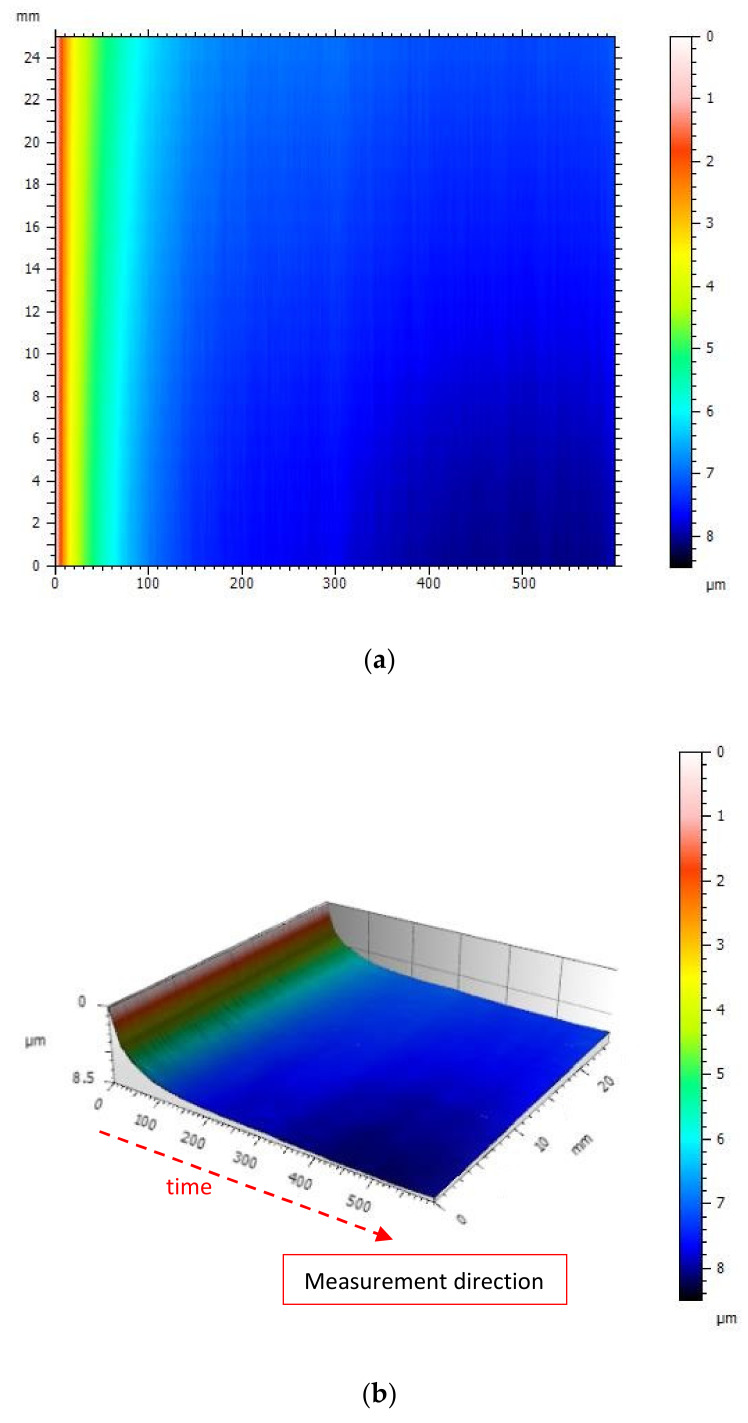
View of the surface generated from the measurements of the same track during heating of the profilometer: (**a**) A color map; (**b**) 3D image.

The illustration (Figure 13) shows the surface generated from the measurements of the same track during heating of the device.

**Figure 14:**
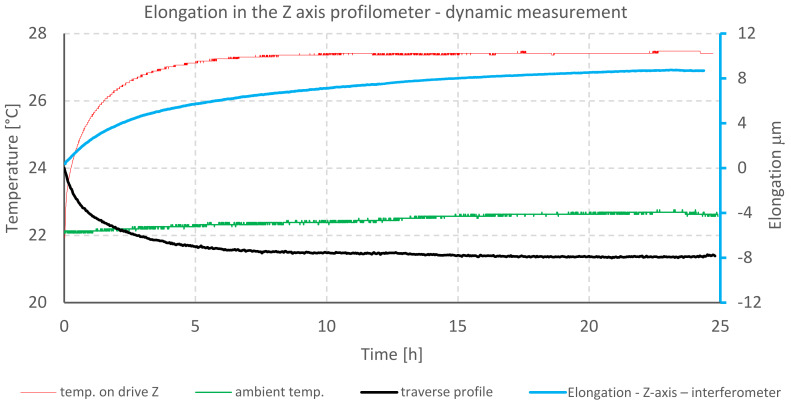
Elongation in *Z*-axis of profilometer A and temperature against time - dynamic measurement.

The graph (Figure 14) shows the correlation between the increase in temperature of the *Z*-axis drive and elongation of the profilometer column. The graph shows the displacement value of the measuring probe, which was measured with the laser interferometer and the measured surface transverse profile.

**Figure 15:**
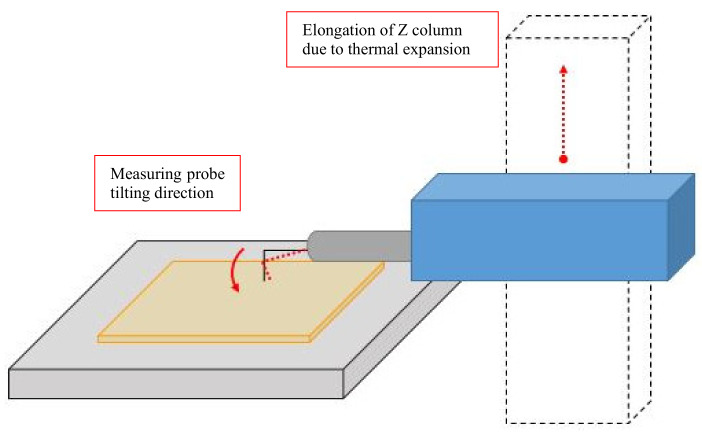
Probe movement due to heating of profilometer column.

The cross-profile curves and the elongation values are inverted. This should be interpreted in such a way that the measuring tip, which was permanently in contact with the specimen, as an effect of elongation (due to the thermal expansion of the Z column), moved below the zero line, and the elongation of the column had a positive value in relation to the measured surface (Figure 15).

The thermal conduction mechanism, concerning heat coming from power supplies or moving elements mounted inside a profilometer, influences the surface topography representation, particularly when changes in temperature take place within a column (Z direction). This results in displacement of the whole drive unit in relation to the base. Thus, the change in geometrical dimension due to the thermal expansibility of the column (elongation and shrinkage) results in the movement of a measuring tip being in contact with a measured surface. This movement is an error, which changes the representation of a surface and gives wrong values of topography parameters, a reason of workpiece malfunctioning and improper classification of manufactured parts. 

The data presented above show the correlation of the temperature increase of the *Z*-axis drive (and the profilometer column) with the value of the *Z*-axis elongation. The maximum displacement of the measuring point placed on the measuring probe in the *Z*-axis reaches 8.3 μm in relation to the initial position, while the temperature of the Z column increased by 5.58 °C. These data correspond to the readings directly from the probe, presented in the form of a transverse profile. The maximum value of the probe displacement was 7.9 μm.

In the second test of the dynamic experiment, the profilometer performed the same profile measurement after thermal stabilization of its drives and structure. This test was intended to indicate differences in the fidelity of surface imaging when the influence of internal heat sources of the profilometer was significantly reduced.

**Figure 16:**
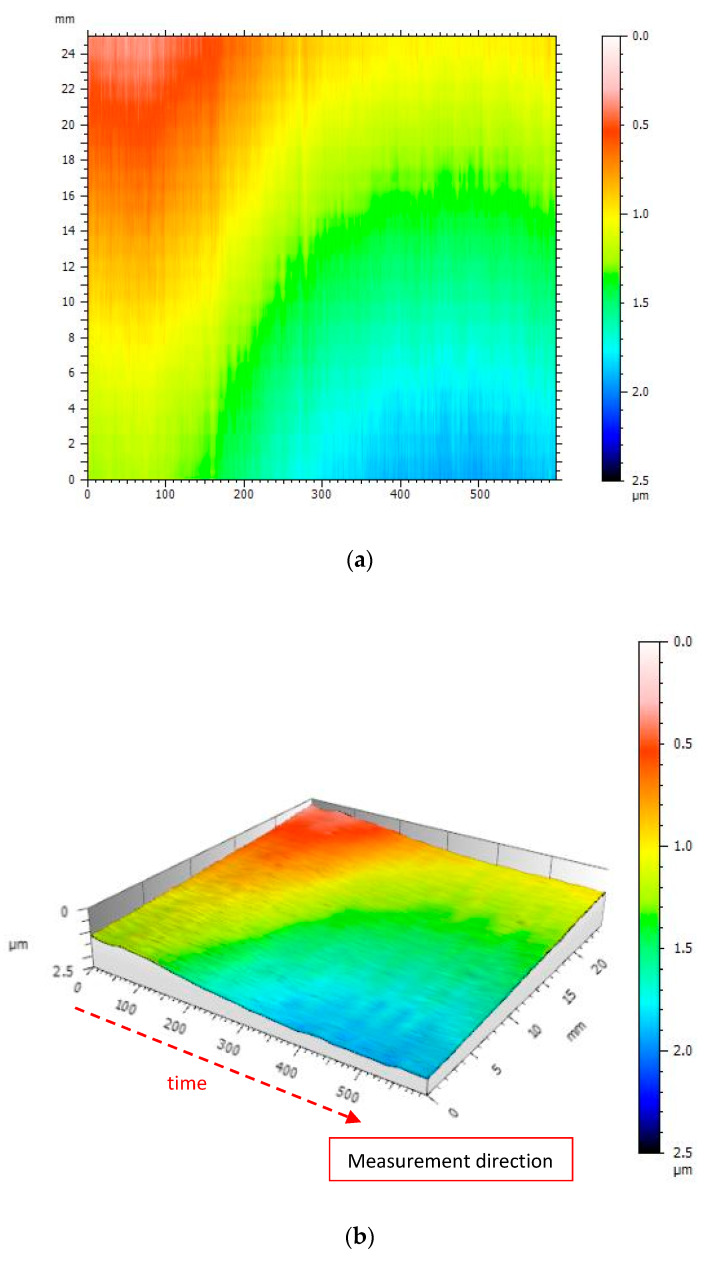
View of the surface generated from measurements of the same profile performed after thermal stabilization of the profilometer: (**a**) A color map; (**b**) 3D view.

The illustration (Figure 16) shows the surface generated from the measurements of the same profile after thermal stabilization of the profilometer.

The generated surface presented in the illustration (Figure 16) shows 90% less noise than in the case of an unstable profilometer. The signal amplitude illustrated in the figure is 0.8 μm, which is about 10 times less than in the case of a thermally unstable profilometer.

It is considered that regardless of the type of drives and electronic components used in a profilometer, if possible, they should be placed outside the profilometer housing so that the heat generated by them would be freely radiated to the environment. Motors should be separated from lead screws/linear guides to reduce heat transfer to essential elements of a measuring device, and connection with lead screws should be made with flexible toothed belts. This solution should protect the profilometer against vibrations coming from the drives while eliminating engine-lead screw clearances and, above all, reduce thermal conductivity between individual components.

## 4. Conclusions

After analyzing results of the research, a direct influence of thermal phenomena on surface imaging errors becomes clearly visible. These errors are especially evident during the initial phase of surface topography measurement, immediately after turning the device on.

There is a relation between the increase in temperature of drives and electronic components installed in contact profilometers (internal heat sources) and the deformation of the device structure in its individual axes. This directly affects the imaging of surface asperities, particularly when the measurement was started immediately after turning the profilometer on.

- The value of elongation in individual axes of the profilometer is different and it very much depends on the construction of the device, type of drives used, and their location. This might indicate that a change in design can limit the influence of thermal disturbances on the measurement results. Thus, it would improve the metrological characteristics of the device.
- The largest value of the displacement of the measuring tip occurs in the direction of the *X*-axis. This value (in the considered cases) reaches 16.2 μm.
- The largest impact on the imaging of the surface topography has the displacement of the probe in the direction of the *Z*-axis. This displacement directly translates into the obtained value of the height of the measured surface.
- The thermal and geometrical stabilization times should be precisely determined before beginning a 3D surface measurement. The stabilization time should be determined individually for a specific type of device in order to make a measurement correctly. Performing thermal stabilization of the tested device has reduced surface imaging errors by 90%.
- The comparison of analyzed constructions and drives of the contact profiler (based on Figure 4, Figure 6 and Figure 8) showed that DC motors working uniformly during the whole measurement are characterized by the best thermal properties. Change in feed should be executed by an electromagnetic clutch.
- Profilometers in which electronic systems and drives were located outside of the device body were characterized by lower values of displacement resulting from thermal deformation than the profilometer with drives inside its structure.
